# The Relationship between Post-COVID Syndrome and the Burden of Comorbidities Assessed Using the Charlson Comorbidity Index

**DOI:** 10.3390/medicina59091583

**Published:** 2023-08-31

**Authors:** Lorenzo Falsetti, Vincenzo Zaccone, Luca Santoro, Silvia Santini, Emanuele Guerrieri, Luca Giuliani, Giovanna Viticchi, Serena Cataldi, Antonio Gasbarrini, Francesco Landi, Angelo Santoliquido, Gianluca Moroncini, on behalf of Gemelli Against Covid-Post-Acute Care Study Group

**Affiliations:** 1 Clinica Medica, Department of Clinical and Molecular Sciences, Marche Polytechnic University, 60126 Ancona, Italy; l.falsetti@staff.univpm.it (L.F.); g.moroncini@staff.univpm.it (G.M.); 2 Internal and Subintensive Medicine, Azienda Ospedaliero-Universitaria delle Marche, 60126 Ancona, Italy; vincenzozaccone@gmail.com; 3 Department of Cardiovascular and Thoracic Sciences, Fondazione Policlinico Universitario Agostino Gemelli IRCCS, 00168 Rome, Italy; angelo.santoliquido@policlinicogemelli.it; 4 Emergency Medicine Residency Program, Marche Polytechnic University, 60126 Ancona, Italy; silviasantini96@gmail.com (S.S.); e.guerrieri93@gmail.com (E.G.); luca.giuliani33@gmail.com (L.G.); 5 Clinica Neurologica, Department of Medical and Surgical Sciences, Marche Polytechnic University, 60126 Ancona, Italy; g.viticchi@staff.univpm.it; 6 Department of Pediatrics, Marche Polytechnic University, 60126 Ancona, Italy; cataldiserena@gmail.com; 7 Department of Medical and Surgical Sciences, Fondazione Policlinico Universitario Agostino Gemelli IRCCS, 00168 Rome, Italy; antonio.gasbarrini@policlinicogemelli.it; 8 Università Cattolica del Sacro Cuore, 00168 Rome, Italy; francesco.landi@policlinicogemelli.it; 9 Geriatrics Department, Fondazione Policlinico Universitario Agostino Gemelli IRCCS, 00168 Rome, Italy

**Keywords:** post-COVID-19, long COVID, post-acute sequelae SARS-CoV-2 infection (PASC), COVID-19, Charlson Comorbidity Index, comorbidities, frailty

## Abstract

*Introduction:* The post-COVID-19 syndrome is a clinical entity characterized by the manifestation of signs and symptoms that develop after the acute phase of COVID-19, which persist for a duration of more than 12 weeks and are not explained by any alternative diagnosis. It has been observed that individuals with pre-existing chronic diseases, including cardiovascular and pulmonary diseases, are at a greater risk of developing post-COVID-19 syndrome. The Charlson Comorbidity Index (CCI) is a useful tool employed to evaluate the burden of comorbidities and predict the prognosis of patients with post-COVID-19 syndrome. The present study aims to assess whether the burden of comorbidities, evaluated using the CCI, correlates with post-COVID-19 syndrome. *Materials and Methods:* Between 21 April 2020 and 15 May 2023, we enrolled all consecutive outpatients with previous COVID-19 admissions to a post-acute day-hospital service three months after a negative SARS-CoV-2 molecular test. We assessed age, sex, BMI, acute COVID-19 and post-COVID-19 signs, and symptoms and calculated CCI according to its current definition. Post-COVID-19 syndrome was defined as the persistence of at least one sign or symptom lasting more than 12 weeks after COVID-19 resolution and not explained by an alternative diagnosis. The relationship between post-COVID-19 and CCI was explored first with the chi-squared test, then with different binary logistic regression models. We considered significant values of *p* lower than 0.05. *Results:* We obtained a cohort of 3636 patients and observed a significant association between the number of post-COVID-19 symptoms and CCI. Patients developing post-COVID-19 were more commonly affected by a greater burden of comorbidities. Patients with at least one CCI point had an increased risk of post-COVID-19 syndrome (OR:2.961; 95%CI: 2.269–3.863; *p* < 0.0001), which increased further for CCI ≥ 4 (OR:6.062; 95%CI: 3.163–11.618; *p* < 0.0001). *Conclusions:* Patients affected by post-COVID-19 show a greater clinical complexity and a larger burden of comorbidities, synthesized by a higher CCI; moreover, a higher CCI seems to correlate with an increasing post-COVID-19 risk, being the presence of ≥1 or ≥4 CCI points associated with a 3-fold and 6-fold increased risk of post-COVID-19 syndrome, respectively.

## 1. Introduction

Post-acute sequelae of COVID-19, also named “post-COVID-19”, represents a clinical syndrome characterized by the onset or persistence of systemic and organ symptoms after the acute phase of a COVID-19 infection, lasting for more than 12 weeks and not explained by an alternative diagnosis [1]. Symptoms generally consist of persistent fatigue, breathlessness, cognitive difficulties (such as brain fog and difficulty concentrating), musculoskeletal pain, sleep disturbances, mood disorders (such as depression and anxiety), and various other symptoms that may involve the respiratory, cardiovascular, gastrointestinal, neurological, dermatological, or other systems [2,3].

Several studies are ongoing to better understand the duration, severity, underlying mechanisms, and appropriate management strategies for individuals experiencing these prolonged symptoms. Several risk factors may contribute to post-COVID-19 syndrome or prolonged symptoms after a COVID-19 infection, such as severity and duration of initial infection, age and sex, immune response, associated disorders, and genetic factors [4].

Some studies have already shown that pre-existing medical conditions, such as obesity, diabetes, cardiovascular disease, lung disease, immunosuppression, and mental health disorders may increase the risk of developing post-COVID-19 syndrome. In particular, patients with cardiac and cardiovascular diseases are more likely to develop post-COVID-19 syndrome [5]. Several authors underlined that both comorbidities and COVID-19 itself worsen endothelial dysfunction, which is currently deemed to be one of the most important determinants in the development of severe COVID-19 and in the persistence of several post-COVID-19 signs and symptoms [6].

The Charlson Comorbidity Index (CCI) is a scoring system used in medical research and in clinical practice to quantify the subject’s clusterization of comorbidities, thus, to assess prognosis [7]; the higher the CCI score, the greater the burden of comorbidities and the higher the predicted risk of mortality. CCI has been validated in several acute and chronic settings [8] and is currently used as an indicator of the clinical complexity of elderly and comorbid patients. People with more comorbidities have a higher risk of developing severe COVID-19 in the acute phase of the disease [9]. However, to our knowledge, no studies have correlated the comorbidities clusterization, assessed using the Charlson Comorbidity Index, with the risk of developing post-COVID-19 syndrome.

Our study aims to define the relationship between the burden of comorbidities, as identified using the CCI, and the risk of developing post-COVID-19 syndrome by analyzing a large and heterogeneous cohort of patients 12 weeks after the resolution of the acute phase of COVID-19 infection.

## 2. Materials and Methods

Our group conducted the current study using a registry called the “Post-acute COVID-19 Day Hospital Unit registry—Fondazione Policlinico Universitario Agostino Gemelli IRCCS di Roma, Italy”, a single-center, prospective, observational registry that included outpatients with a previous COVID-19. In the timeframe between 21 April 2020 and 15 May 2023, the working group enrolled all the consecutive outpatients with previous COVID-19 three months after a negative SARS-CoV-2 molecular test. The enrolled patients did not undergo SARS-CoV-2 genotyping but the time period analyzed comprised all the viral strains observed in Italy in the different COVID-19 waves (wild-type, alpha, beta, delta, and omicron strains). Each patient underwent an exhaustive medical evaluation in the post-acute phase of the disease through a comprehensive clinical and multidisciplinary assessment, as already described in detail in another publication by our working group [10].

We paid special attention to the COVID-19-related history of each patient, asking the enrolled subjects to describe their past and present symptoms and signs of COVID-19, the treatment they received for the disease, and the signs and symptoms that persisted at the follow-up visit. We also focused our attention to each subject’s comorbidities, synthesizing them into the Charlson Comorbidity Index (CCI), which was calculated according to its original definition [7], as shown in Table 1.

### 2.1. Ethical Approval

This study was approved by the ethics committee of the University Cattolica del Sacro Cuore in Rome (protocol ID number: 003220/20) and was performed in accordance with the Declaration of Helsinki [11]. All patients provided their written consent to participate.

### 2.2. Patients

For each patient, we assessed age, sex, body mass index (BMI, kg/m^2^), each COVID-19 and post-COVID-19 sign and symptom, the absolute number of post-COVID-19 signs and symptoms and calculated CCI. Post-COVID-19 syndrome was defined as the persistence of at least one sign or symptom lasting for more than 12 weeks after COVID-19 resolution. 

The working group considered the following signs or symptoms at the follow-up from the beginning of the study: fever, fatigue, cough, diarrhea, headache, anosmia, dysgeusia, red eyes, low or blurred vision, syncope, vertigo, joint pain, skin lesions, sicca syndrome, Raynaud’s phenomenon, myalgia, dyspnea, chest pain, sore throat, sputum, rhinitis, lack of appetite, tinnitus, heartburn, sleep disturbances, brain fog and cognitive disturbances, and tingling. All the signs or symptoms evaluated in this study were regarded as part of the post-COVID-19 syndrome only if—at the end of the multidisciplinary workout—no other cause of the sign or symptom arose. 

### 2.3. Statistical Analysis

Age, BMI, CCI, and the absolute number of post-COVID-19 symptoms were treated as continuous variables. Sex, presence of post-COVID-19 syndrome, and each COVID-19 and post-COVID-19 sign or symptom were treated as binary variables. BMI was recoded into a three-level categorical variable considering the cutoffs suggested by the World Health Organization (normal weight < 25 kg/m^2^; overweight 25–30 kg/m^2^; obese > 30 kg/m^2^) [12]. CCI was also recoded into two dichotomous variables (in particular, we adopted two cut-offs: ≥1 and ≥4) and into one categorical variable (adopting four categories: CCI = 0; CCI = 1–2; CCI = 3–4; and CCI ≥ 5) as suggested by the literature [13].

Continuous variables were tested for normality with the Kolomogorov–Smirnov test. Normally distributed variables were presented as mean and standard deviation (SD) and compared with a t-test or ANOVA. Non-normally distributed variables were presented as median and interquartile range (IQR) and compared with the Mann–Whitney U test or Kruskal–Wallis H test. Categorical and dichotomous variables were presented as number and percent and compared with the chi-squared test.

The association between variables was explored using Pearson’s bivariate test, and the Pearson’s coefficient was used as a measure of a linear association between variables. The relationship between post-COVID-19 syndrome and CCI (both in categorial and dichotomous form) was explored using a chi-squared test. The odds ratio (OR) of developing a post-COVID-19 syndrome according to CCI was assessed with binary logistic regression models considering post-COVID-19 syndrome as a dependent variable and CCI—in dichotomous or in continuous form—as the main predictor. We analyzed both uncorrected and corrected methods that considered sex and BMI as covariates (age was not added to the model to reduce multicollinearity, since it was already considered in CCI). We considered significant values of *p* lower than 0.05. The analysis was performed with SPSS 13.0 (SPSS, Chicago, IL, USA) for Windows Systems.

## 3. Results

We obtained a cohort of 3636 patients, of which, 3394 developed a post-COVID-19 syndrome at the control performed after the acute phase of disease. The baseline characteristics of the sample are described in Table 2.

CCI results associated, at Pearson’s bivariate test, with post-COVID-19 syndrome (Pearson Correlation = 0.112; *p* < 0.0001), age (Pearson Correlation = 0.809; *p* < 0.0001), sex (Pearson Correlation = −0.110; *p* < 0.0001), and BMI (Pearson Correlation = 0.162; *p* < 0.0001). Post-COVID-19 syndrome resulted associated, at Pearson’s bivariate test, with CCI (Pearson Correlation = 0.112; *p* < 0.0001) and BMI (Pearson Correlation = 0.044; *p* = 0.01). 

**Figure 1:**
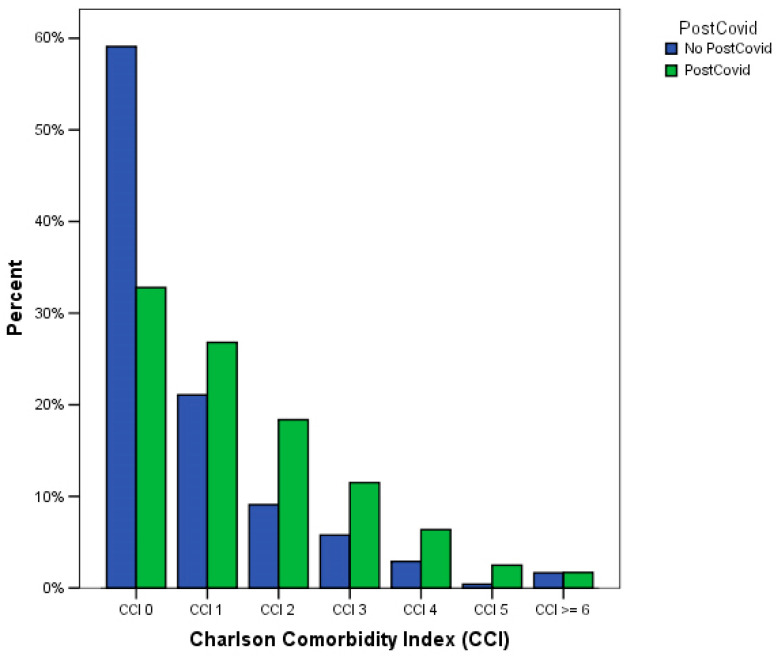
Relationship between post-COVID-19 and Charlson Comorbidity Index (*p* < 0.0001 at chi-squared test).

Patients developing post-COVID-19 syndrome were more commonly affected by a greater burden of comorbidities (*p* < 0.0001, chi-squared test), as shown in Table 3 and Figure 1. Similarly, when considering the CCI as a categorical variable, we observed a significant decrease in the proportion of patients free from post-COVID-19 at 12 weeks (from 11.4% at CCI = 0 to 3.4% at CCI ≥ 5, *p* < 0.0001 at chi-squared test). Moreover, patients who did not develop a post-COVID-19 syndrome had a significantly lower median value of CCI (0 [1]) than patients who developed this complication (2 [2], *p* < 0.0001 at Mann–Whitney U test). 

**Figure 2:**
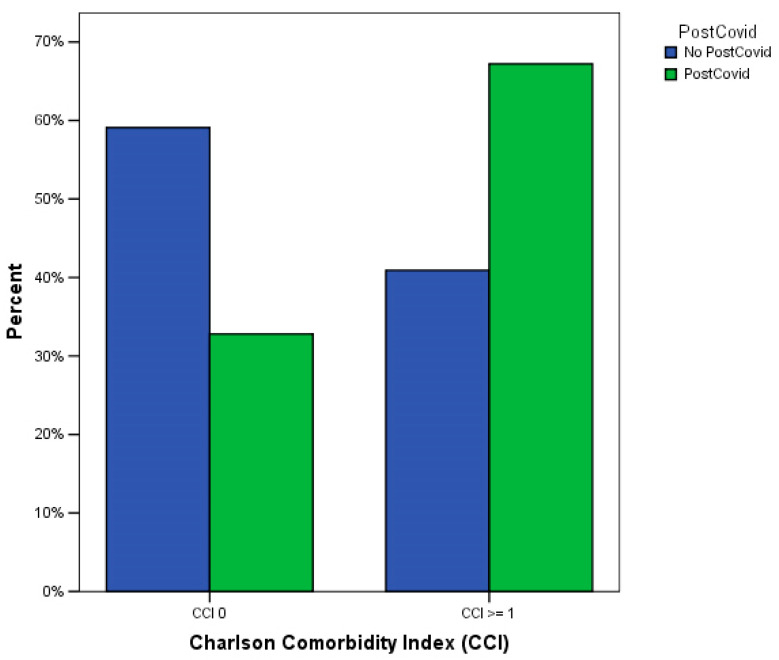
Relationship between at least one post-COVID-19 symptom and at least one point at Charlson Comorbidity Index (*p* < 0.0001 at chi-squared test).

Patients with at least one CCI point developed more commonly a post-COVID-19 syndrome than patients with no comorbidities (*p* < 0.0001, chi-squared test), as shown in Table 4 and Figure 2.

In the first binary logistic regression analysis, the occurrence of post-COVID-19 syndrome was the main dependent variable, while the binary CCI variable (adopting a cutoff of CCI ≥ 1) was the independent variable. In this model, the presence of at least one comorbidity at CCI was significantly associated with an increased risk of post-COVID-19 syndrome (OR: 2.961; 95%CI: 2.269–3.863; *p* < 0.0001). Modifying the cutoff of the binary CCI variable at ≥4 in the same model, we observed that the risk of developing a post-COVID-19 syndrome further increased (OR: 6.062; 95%CI: 3.163–11.618; *p* < 0.0001). 

In the second binary logistic regression analysis, the occurrence of post-COVID-19 syndrome was the main dependent variable, while the continuous CCI variable was the independent variable. In this model, a one-unit increase in CCI was significantly associated with a significantly increasing risk of post-COVID-19 syndrome (OR: 1.503; 95%CI: 1.331–1.697; *p* < 0.0001). We also observed similar results when adding sex and BMI as covariates to the binary logistic models, as shown in Table 5. In these two models, we also observed that female sex and higher BMI categories were associated with an increased risk of developing post-COVID-19 syndrome.

## 4. Discussion

In this prospective observational study, we have enlightened a strong correlation between the burden of comorbidities of patients with COVID-19 and the subsequent onset of post-COVID-19 syndrome; indeed, in our cohort, patients with post-COVID-19 syndrome were more likely to have comorbidities and, conversely, the presence of comorbidities significantly increased the likelihood of developing post-COVID-19 syndrome.

Previous studies have already shown that COVID-19, caused by the SARS-CoV-2 virus, has a significant impact on individuals with pre-existing comorbidities, in particular, diabetes, hypertension, obesity, heart disease, chronic lung disease, and immunocompromised states, among others [14]. With this study, we aimed to underline the importance of clustering different chronic diseases performed with a score already validated in several acute and chronic settings, such as the CCI. By considering most of the commonly assessed comorbidities, the Charlson Comorbidity Index can more precisely assess the load of chronic disorders at the patient’s level, thus, describing synthetically the clinical complexity of the subject.

The relationship between the burden of comorbidities and COVID-19 is complex and multidimensional. Some comorbidities can weaken the immune response, making it less effective at clearing the virus; individuals with diabetes [15] or autoimmune disorders [16] may have a reduced immune response that makes them more prone to severe COVID-19. On the other side, other disorders are associated with chronic inflammation, and an overactive immune response can trigger a cytokine storm during COVID-19 infection; a disproportionate immune response can cause widespread inflammation and end-organ damage leading to the most severe forms of COVID-19 [17]. In other cases, COVID-19 can also exacerbate the symptoms and complications of pre-existing comorbidities [18]. Therefore, people with comorbidities are more likely to develop severe COVID-19 if they become infected; COVID-19 may lead to more severe respiratory complications, higher rates of hospitalization, and an increased risk of mortality in these populations [19].

Post-COVID-19 is a condition in which individuals experience persistent symptoms or develop new health problems that persist for weeks or months after the acute phase of COVID-19 [1]. The emergence of new variants of SARS-CoV-2 has raised concerns about their differential impact on post-COVID-19 symptoms. Recent evidence suggests that these variants may be associated with different phenotypes, which can be attributed to the varying virus–host interactions [20,21]. This could explain the very wide variety of post-COVID-19 symptoms’ clustering observed during the COVID-19 pandemic. Further research is necessary to fully understand the implications of these variants on post-COVID-19 clinical phenotype. Some studies suggest that people with specific comorbidities, such as diabetes or autoimmune diseases, may be more likely to develop post-COVID-19 syndrome [22]. Several studies have already shown that frail and comorbid patients hospitalized for COVID-19 are more likely to continue experiencing health problems and adverse outcomes after their initial recovery [13]. Despite a growing body of evidence, the relationship between post-COVID-19 syndrome and the patient’s clinical complexity is still not fully understood and represents an area of ongoing research. A potential direction in this topic is represented by the assessment of the clinical post-COVID-19 phenotype according to the interactions between patients’ characteristics, such as the burden of comorbidities, immune and endothelial function, genetic determinants, and viral genotype.

Preliminary studies have shown that a high CCI correlates with reduced physical performance indices in patients at one-year follow-up after COVID-19. After discharge from the hospital, COVID-19 survivors with higher CCI scores tend to have worse 6 min walk test scores at one-year follow-up, suggesting that a higher CCI score is associated with reduced physical functioning and overall decreased health outcomes in these individuals [23]. 

In our study, we showed that a higher CCI score, representing a greater clusterization of chronic diseases, appears to correlate with a greater likelihood of post-COVID-19 syndrome. This suggests that individuals with more pre-existing medical conditions may be more likely to experience a wider range of systemic symptoms after recovery from the acute phase of COVID-19. Even the presence of just one point at CCI is associated with a 3-fold risk increase in post-COVID-19 syndrome; in this sense, a higher age, or the presence of having even an mild, pre-existing medical condition significantly increases the likelihood of experiencing prolonged symptoms and complications after COVID-19 recovery. In addition, further increases in CCI spread the risk of post-COVID-19; in fact, in our study, individuals with a CCI ≥ 4 were six times more likely to develop post-COVID-19 syndrome than those with a lower CCI score.

There are several speculative explanations for the associations found in our study. It is reasonable that individuals with a higher burden of comorbidities, experiencing a more severe acute illness [14], could develop post-COVID-19 syndrome. Furthermore, comorbidities could exacerbate inflammatory response and immune dysfunction [17], leading to a more prolonged and complicated recovery from COVID-19. Furthermore, several studies have shown that the occurrence of specific metabolic and cardiovascular comorbidities can lead to a greater extent of endothelial dysfunction, which is further worsened by the acute COVID-19 infection. It has been found that endothelial dysfunction plays a pivotal role in the development of many of the immediate complications associated with COVID-19, including acute cardiovascular events and thrombosis. Additionally, it is also linked to the onset of various post-COVID-19 signs and symptoms [6,24]. Finally, patients with pre-existing comorbidities may have lower functional organ reserve and, therefore, be more likely to develop post-COVID-19 syndrome. In general, the presence of comorbidities has been already shown to influence the acute and post-acute progression of other pathological conditions [8,25].

Our findings highlight the importance of also considering the subject’s comorbidities and their clusterization when assessing the risk of developing post-COVID-19. The results of our study, if confirmed in other cohorts, suggest that assessing the comorbidity load to evaluate the potential impact on the development of post-COVID-19 syndrome could represent an additional resource for the practicing clinician. Indeed, using the CCI score as a screening tool in patients with COVID-19 can help healthcare professionals identify those who may require more comprehensive care and resources, potentially improving their overall outcomes and their quality of life in the long term. A very high CCI score suggests the presence of a significant burden of comorbidities. Such a clinical complexity could prompt clinicians to take additional precautions, offer specialized care, or closely monitor the patient’s health during the one-year follow-up period after COVID-19 and beyond.

It is important to underline that post-COVID-19 syndrome is a complex and multilayered condition, and its development and severity have a great inter-individual variation. Further research is needed to understand the exact relationship between post-COVID-19 syndrome and comorbidities.

### Study Limitations

The main limitation of this study is related to the typology of patients effectively enrolled and followed-up; older and frail subjects experiencing the most severe forms of COVID-19 could have been lost at the follow-up and could be under-represented in the analyzed cohort. Similarly, younger patients affected by milder forms of COVID-19 could have been over-represented in the population under analysis. Moreover, the creation of a service specifically designed to assess post-COVID-19 sequelae could have increased the number of symptomatic subjects in our cohort for selection bias. The absence of the SARS-CoV-2 genotype did not allow us to describe the phenotype of the post-COVID-19 syndrome according to this characteristic. Some symptoms, such as fatigue, are very vague and common and could have contributed to an overdiagnosis of post-COVID-19 syndrome. However, several cohort studies estimate the prevalence of post-COVID-19 syndrome between 35 and 85%, depending on the severity of the acute phase of the disease [26]. Of note, since a very wide variety of signs or symptoms has been described as a part of the post-COVID-19, it is very difficult to extensively analyze all the components of this heterogeneous clinical entity and to exactly assess its real prevalence according to its current definition. However, by analyzing the most frequently described manifestations of post-COVID-19, we were able to obtain a good estimate of the prevalence of each symptom. Last, albeit common, we did not retrieve any information regarding the occurrence of deep vein thrombosis, pulmonary embolism, and acute vascular events after the acute phase of the infection, focusing mainly on the development of subacute signs and symptoms.

## 5. Conclusions

The burden of comorbidities and the subject’s clinical complexity, measured using CCI, is associated with an increased risk of post-COVID-19 syndrome. Increasing CCI seems to be correlated with an increased risk of post-COVID-19, being the presence of ≥1 or ≥4 CCI points associated with a 3-fold and 6-fold increased risk of post-COVID-19 syndrome, respectively.

## Figures and Tables

**Table 1:** The Charlson Comorbidity Index.

Item	Points
Age <50 years 50–59 years 60–69 years 70–79 years ≥80 years	0 +1 +2 +3 +4
Myocardial infarction	+1
Chronic heart failure	+1
Peripheral vascular disease	+1
Cardiovascular event or TIA	+1
Dementia	+1
COPD	+1
Connective tissue disease	+1
Peptic ulcer disease	+1
Liver disease None Mild Moderate-to-severe	0 +1 +3
Diabetes None or diet-controlled Uncomplicated End-organ damage	0 +1 +2
Hemiplegia	+2
Moderate-to-severe CKD	+2
Solid tumor None Localized Metastatic	0 +2 +6
Leukemia	+2
Lymphoma	+2
AIDS	+6

Legend: AIDS = acquired immunodeficiency syndrome; COPD = chronic obstructive lung disease; CKD = chronic kidney disease; TIA = transient ischemic attack.

**Table 2:** Baseline characteristics of the sample.

Variable	Mean	
Age (mean, ±SD), years	53.7 ± 14.6 years	
BMI (mean, ±SD), kg/m^2^	26.2 ± 4.90 kg/m^2^	
Male sex (n, %)	1749 (48.1%)	
Charlson Comorbidity Index (median, IQR)	1 [2]; range: 0–10	
COVID-19 signs and symptoms (median, IQR)	7 [7]; range: 0–22	
Charlson Comorbidity Index (n, %) 0 points ≥1 point	1265 (34.8%) 2371 (65.2%)	
Post-COVID-19 syndrome (n, %) No Yes	242 (6.7%) 3394 (93.3%)	
Post-COVID-19 signs and symptoms (median, IQR)	3 [4]; range: 0–22	
COVID-19 and post-COVID-19 signs and symptoms (*)		
	COVID-19	post-COVID-19
Fever (n, %)	2718 (74.8%)	116 (3.2%)
Fatigue (n, %)	2792 (76.8%)	2354 (64.7%)
Cough (n, %)	2025 (55.7%)	476 (13.1%)
Diarrhea (n, %)	867 (23.8%)	281 (7.7%)
Headache (n, %)	1726 (47.5%)	747 (20.5%)
Anosmia (n, %)	1439 (39.6%)	547 (15.0%)
Dysgeusia (n, %)	1444 (39.7%)	468 (12.9%)
Red eyes (n, %)	613 (16.9%)	230 (6.3%)
Low or blurred vision (n, %)	566 (15.6%)	598 (16.4%)
Syncope (n, %)	220 (6.1%)	39 (1.1%)
Vertigo (n, %)	758 (20.8%)	428 (11.8%)
Joint pain (n, %)	1859 (51.1%)	1071 (29.4%)
Skin lesions (n, %)	320 (8.8%)	244 (6.7%)
Sicca syndrome (n, %)	639 (17.6%)	347 (9.5%)
Raynaud’s phenomenon (n, %)	65 (1.8%)	54 (1.5%)
Myalgia (n, %)	1963 (54.0%)	1043 (28.7%)
Dyspnea (n, %)	2118 (58.3%)	1904 (52.3%)
Chest Pain (n, %)	1168 (32.1%)	594 (16.3%)
Sore throat (n, %)	1075 (29.6%)	200 (5.5%)
Sputum (n, %)	592 (16.3%)	218 (6.0%)
Rhinitis (n, %)	952 (26.2%)	263 (7.2%)
Lack of appetite (n, %)	1238 (34.0%)	240 (6.6%)
Tinnitus (n, %)	198 (5.4%)	175 (4.8%)
Heartburn (n, %)	271 (7.4%)	281 (7.7%)
Palpitations (n, %)	473 (13.0%)	369 (10.1%)
Sleep disturbances (n, %)	506 (13.9%)	502 (13.8%)
Brain fog and cognitive disturbances (n, %)	447 (12.3%)	726 (20.0%)
Tingling (n, %)	277 (7.6%)	330 (9.1%)

Legend: BMI = body mass index; IQR = interquartile range; SD = standard deviation; (*) some patients’ signs or symptoms were dropped at the end of follow-up since an organic cause emerged during the multidisciplinary assessment; in other subjects, there were missing cases: we report the percentage of the positive patients in the cohort excluding the missing/dropped cases.

**Table 3:** Relationship between post-COVID-19 and Charlson Comorbidity Index (*p* < 0.0001 at chi-squared test).

Charlson Comorbidity Index	Post-COVID-19 Syndrome					
	No Post-COVID-19 Syndrome			Post-COVID-19 Syndrome		
	n	c	r	n	c	R
0 points	143	59.1%	11.4%	1107	32.6%	88.6%
1 point	51	21.1%	5.3%	907	26.7%	94.7%
2 points	22	9.1%	3.4%	620	18.3%	96.6%
3 points	14	5.8%	3.4%	396	11.7%	96.5%
4 points	7	2.9%	3.0%	223	6.6%	96.9%
5 points	1	0.4%	1.2%	84	2.5%	98.8%
≥6 points	4	1.7%	6.5%	57	1.7%	93.4%

Legend: n = absolute number; c = column percentage; r = row percentage.

**Table 4:** Relationship between at least one post-COVID-19 symptom and at least one point at Charlson Comorbidity Index (*p* < 0.0001 at chi-squared test).

Charlson Comorbidity Index	Post-COVID-19 Syndrome	
	No Post-COVID-19 Syndrome	Post-COVID-19 Syndrome
0 points	143 (59.1%)	1107 (32.6%)
≥1 point	99 (40.9%)	2287 (67.4%)

**Table 5:** Binary logistic regression analysis results (corrected models).

Model 1	Risk of Post-COVID-19 Syndrome		
Variable	Odds Ratio	95%CI	*p*
Binary CCI (≥1)	2.197	1.621–2.977	0.0001
Female Sex	1.831	1.410–2.379	0.0001
Categorial BMI	2.071	1.590–2.698	0.0001
Model 2	Risk of post-COVID-19 syndrome		
Variable	Odds Ratio	95%CI	*p*
Binary CCI (≥4)	6.062	3.163–11.618	0.006
Female Sex	1.349	0.969–1.877	0.076
Categorial BMI	1.829	1.396–2.397	0.0001
Model 3	Risk of post-COVID-19 syndrome		
Variable	Odds Ratio	95%CI	*p*
Continuous CCI	1.201	1.053–1.368	0.006
Female Sex	2.630	2.097–3.299	0.0001
Categorial BMI	2.838	2.239–3.597	0.0001

Legend: BMI = Body Mass Index; CCI = Charlson Comorbidity Index; CI = Confidence Interval.

## Data Availability

The data presented in this study are available on request from the corresponding author. The data are not publicly available due to privacy issues.
